# 

**Published:** 1874-10

**Authors:** 


ESTABLISHED IN 1855.

VOLUME XII. | OCTOBER—1874. | NUMBER 7.

### ATLANTA

### Medical and Surgical Journal.

MONTHLY: THREE DOLLARS PER ANNUM, IN ADVANCE.

ROBERT BATTEY, M.D.,

EDITOR.

WM. ABRAM LOVE, M.D., V. H. TALIAFERRO, M.D.,

ASSOCIATE EDITORS.

*PAX ET SCIENTIA, SED VERITAS SINE TIMORE.*

ATLANTA, GEORGIA:

